# Meetings of Societies

**Published:** 1897-12

**Authors:** 


### MEETINGS OF SOCIETIES.

### Bristol Medico-Chirurgical Society.

*Annual Meeting, October 13th*, 1897.

Dr. A. E. Aust Lawrence, President, in the Chair.

After a vote of thanks to the retiring President (Dr. A. E. Aust Lawrence) had been unanimously passed, Dr. J. E. Shaw (the President-elect) took the Chair, and gave his introductory address (see page 301), for which a cordial vote of thanks was given him.

The Honorary Secretary (Mr. J. Paul Bush) read his report, in which he stated that the members of the Society now numbered 134. He referred to the lamented death of Mr. Greig Smith, and mentioned that a fitting memorial had been started to perpetuate his memory.[1]

1 Mr. Paul Bush and Dr. James Swain, who are the Honorary Secretaries of the fund being raised for the purpose, will be glad to receive donations from any who would like to contribute.

The Editorial Secretary (Dr. Bertram M. H. Rogers), in his report, said that the cost of the *Journal* was constantly increasing, partly from its larger size and partly from the numerous illustrations that were introduced, which latter, while making the *Journal* more interesting, could not be done without additional outlay. He begged all members of the Society to assist him in obtaining new subscribers, for he regretted to say that at least half the medical men of the city were still neither members of the Society nor subscribers to the *Journal.*

The Honorary Librarian read his annual report (see page 367).

All these reports were adopted, and the readers of them were thanked for their services.

The following officers were chosen for the ensuing year :—President-elect; Dr. R. Roxburgh: Honorary Secretary; Mr. J. Paul Bush: Honorary Librarian; Mr. L. M. Griffiths: Members of Committee; Dr. J. Michell Clarke, Mr. J. Dacre, Mr. W. H. Harsant, Dr. Bertram M. H. Rogers, Mr. G. Munro Smith, and Dr. H. Waldo: Representatives of the Society on the Committee of the Bristol Medical Library; Mr. J. Paul Bush, Mr. L. M. Griffiths, and Dr. G. Parker.

---

*November 10th*, 1897.

Dr, J. E. Shaw, President, in the Chair.

Dr. Michell Clarke showed a sailor, a native of Calcutta, who came to the General Hospital for œdema of the feet, and with a trace of albumen in the urine, but with no other symptoms. There was no chyluria. Examination of the blood showed the presence of filaria nocturna in small number; they were absent from the blood during the day. Specimens of the living and dead filaria were shown under the microscope.

Dr. E. C. Williams showed two cases fairly typical of the class of case which could be expected to derive benefit from the Tallerman-Sheffield treatment. (1) A boy, aged 8 years, was admitted into the Children's Hospital with a stiff left knee-joint and inability to flex the knee and walk. After six baths it was possible to slightly flex the knee, and now, after twenty-five baths, he is able to walk. (2) A girl, aged 17 years, with rheumatoid arthritis, with considerable effusion into the finger-joints; the right elbow was flexed, and it was impossible for her to dress herself. After six baths the effusion was gradually absorbed. Now, after thirteen baths, the patient is able to dress herself. The bath is generally given from forty-five minutes to one hour, and the temperature of the bath varies from 260° to 300° F. The body temperature of the patient is raised 1° or 2° F., and there is profuse sweating. The most satisfactory cases for the bath are those in which there are no organic joint changes. It would also probably be useful in acute gout.

Mr. C. A. Morton showed: (1) A large gallstone which he had removed from the common duct of a woman 72 years of age (see page 317). (2) A renal calculus, and the skiagram which revealed its presence more than a week after all the symptoms of calculus in the kidney had ceased, and when otherwise it might have been thought that the stone had been passed. The stone had been successfully removed from the kidney by operation.—Dr. James Swain referred to the large stones which were capable of entering into and passing along the common duct, and showed a biliary calculus composed of 90 per cent. of cholesterine which he had removed from a patient over 70

years of age, with intestinal obstruction. The calculus weighed 208 grains. The patient had had jaundice nine or ten years before, and twelve months before had had an attack of obstruction, from which she recovered spontaneously after passing a calculus weighing 114 grains. —Dr. E. MARKHAM SKERRITT remarked that gallstones sometimes caused fever of a continuous type, extending over some days, commonly ushered in by a rigor, and marked by considerable exacerbation and remissions, the attacks occurring independently of suppuration.—Mr. C. W. J, BRASHER asked what exposure to the Röntgen rays was given to the oxalate calculus.—Dr. MICHELL CLARKE remarked that the intermittent pyrexia and other symptoms led him to believe that the patient when under his care was suffering from gallstones: whether the cause of the intermittent pyrexia would be found in suppuration in the ducts or not seemed doubtful. An operation was recommended, and she was transferred to Mr. Morton.—Dr. T. FISHER referred to a case of a calculus impacted in the common bile-duct, recently seen in the *post-mortem* room of the Bristol Royal Infirmary, in which, although the common bile-duct and large divisions were quite healthy, there was suppuration in the smaller divisions, and small abscesses were scattered throughout the liver. He pointed out that at the time of operation in such a case the surgeon might have supposed that no suppuration was present. The sequence of Mr. Morton's case proved that such serious complication was not present there, but it was possible that in such cases the first stage was in existence; that is to say, that micro-organisms were present in the retained mucus, giving rise to poisonous toxins. The relief of this retained secretion and the micro-organisms led to immediate fall of temperature.—Mr. MORTON, in reply, said that the temperature in his case of gallstones was very interesting, as it was quite unlike the temperature usually present in ball-valve obstruction of the common duct by gallstones, so clearly described by Osler.[1] In this condition the temperature closely resembles ague, and there is a period of remission of days or weeks, whereas in the case now related there had been a nightly rise of considerable degree, which had suggested the presence of suppurative cholangitis. As Dr. Fisher had said, the speedy convalescence of the patient negatived the possibility that abscesses were present in the liver. In addition to the theory mentioned by Dr. Fisher that the temperature in cases such as Osler described, was due to the formation of some ferment by organisms present in the retained bile, was the theory that the temperature was a reflex nervous phenomenon, and this theory he was himself inclined to accept rather than the other. He was not present when the skiagram showing the renal calculus was taken, and had not heard what the length of exposure had been.

Mr. MUNRO SMITH showed a recent specimen of a tumour, probably fibroid, growing in the mesentery of a fowl.

Dr. F. H. EDGEWORTH read a paper on "Some Diseases occurring in Men who Worked in a Sewer." (This and the discussion which followed it will be printed in a future number.)

Mr. MUNRO SMITH reported a case of cancrum in the ear in a child two years of age. The disease began as an ulcer of the auricle, and gradually extended until a large cavity was excavated leading down to and exposing the condyle of the jaw, the mastoid process and neighbouring parts. The bones exposed were necrosed. The child died from thrombosis of the lateral sinus and meningitis. Dr. Symes made cultures from the rapidly growing ulcer during life, and from the

[1] *Lancet*, 1897, i., 1319.

longitudinal sinus after death. He procured colonies of a rod-shaped bacillus corresponding to that described by Schimmelbusch as occurring in cases of noma.—Dr. T. FISHER said that it might be worth mentioning that this was the third case of thrombosis of cerebral vessels associated with some kind of bacterial infection that had been seen in the *post-mortem* room of the Infirmary during the past few months. One was thrombosis of the basilar artery associated with diphtheria, another thrombosis of the superior longitudinal sinus occurring with membranous tonsillitis, in which, however, diphtheritic bacilli were not found.—Dr. H. ELWIN HARRIS mentioned nine cases of cancrum oris following broncho-pneumonia occurring in a children's ward at an Infirmary. All the cases occurred after admission and in rapid succession, the whole "epidemic" being over in four to six weeks. Subsequently a mushroom bed was discovered immediately under the ward, and he suggested that this might possibly have had something to do with the outbreak, as after its removal no further cases occurred, —Dr. D. S. DAVIES said, with regard to the cases mentioned by Dr. Harris, that after the first case steps should have been taken to disinfect the ward.

Dr. J. O. SYMES gave a demonstration of Widal's reaction for the diagnosis of enteric fever. Two microscopic preparations of typhoid bacilli were exhibited, one to which normal serum had been added, showing the bacilli actively motile and separate; and the second, to which serum from a case of enteric fever had been added, showing the bacilli motionless and clumped. The *technique* of the test was described. Failure to get a reaction may be regarded as tolerably certain evidence that the disease is not enteric fever. Characteristic clumping has occasionally resulted from the addition of serum taken from persons suffering from other diseases; *e.g.* malaria, endocarditis, endometritis. The margin of error is not greater than 10 or 12 per cent. Although the reaction has been obtained as early as the fourth or fifth day of the disease, it is not usually to be got until the end of the first week. It persists from four to six months, but has been obtained after intervals of four and eight years. Of thirty cases tested by Dr. Symes during the present outbreak of enteric fever, twenty-one gave positive reactions in less than half an hour. All of these have since proved clinically to be enteric cases. Three cases gave a positive reaction in less than an hour and a half. One has since proved clinically to be a case of enteric fever, the second was obtained from a child in an infected house who had a brief febrile attack, and the third from a child in whom nothing was detected beyond pyrexia of a few days' duration. Six cases gave no reaction. One of these was from a person in normal health, and two from persons in infected households who suffered from slight indisposition, but neither of whom at any time showed symptoms of enteric fever. The three remaining negative results were probably due to errors in *technique*. In two of the cases a positive result was obtained by the Medical Officer of Health, and in the third a positive reaction was obtained a few days later. All three subsequently were clinically diagnosed as enteric fever.—Dr. D. S. DAVIES said that during the past month the unfortunate milk epidemic had given himself and his colleague, Dr. Heaven, an unusual experience of the Widal-Grünbaum serum test in enteric fever. During this period they had made no less than 152 examinations, most of them within the preceding ten days; of these only twenty cases showed negative results. Clinical observation had now confirmed the majority of the results; many of the cases were clinically uncertain when submitted for examination. It was too early to be able to fully analyse the results side by side with the clinical development, but he felt little doubt that the reaction was of very great value in confirming the diagnosis of enteric fever, and that the percentage of actual error would prove to be very small. The outfits supplied by the Health Department were exhibited, and some of the more typical methods of clumping observed were explained, also the appearances common in "false" clumping. He deprecated the attempt to utilise this test at the bedside, as, although the process of observation was simple enough in theory, it required, for scientific accuracy, the appliances of a laboratory, and a certain trained skill in the manipulation of living pathogenic organisms, and the observation of results. At a later date he hoped to bring before the Society the completed results of his observations, and he was glad to think that this work of the Health Department had been of some service to the local members of the profession in the elucidation of a difficult and, at first, obscure epidemic.—Dr. B. M. H. ROGERS said that it was at present impossible to say how far the test was an absolutely trustworthy one; one case of his in which Dr. Davies had got no reaction subsequently developed into a typical case of enteric fever.—Mr. HANCOCKE WATHEN said that in his experience the test had been of great assistance in determining the diagnosis of doubful cases.

### Plymouth Medical Society.

*Annual Meeting, October 13th,* 1897.

Mr. J. H. S. MAY, President, in the Chair.

The following officers were elected for the ensuing session:—President; Dr. C Aldridge: Hon. Treasurer; Mr. J. E. Square: Hon. Librarian; Mr. C. E. Russel Rendle: Hon. Sec.; Mr. W. L. Woollcombe.

After the meeting 29 members and guests dined together.

*Saturday, October 23rd,* 1897.

Dr. C. ALDRIDGE, in the Chair.

Mr. J. ROLSTON showed: (1) A man, aged 66, who five months ago presented himself on account of diplopia of one week's duration. He had paralysis of right external rectus; high-tension pulse; urine, sp. gr. 1020, acid, no albumen. He now had commencing paresis of left external rectus. No changes in optic discs. Mr. Rolston suggested aneurysmal dilatation of some vessels at base as the probable cause. (2) A child, aged 11 months, with a large hairy mole of left side of forehead, eyebrow, and half upper eyelid, covering an area of about 4 in. by 3 in. The hair was long, silky and dark.—Mr. ALDOUS suggested a trial of Lewis Jones's electrical method.—Mr. C. E. RUSSEL RENDLE, sharp spooning in segments.—Mr. LUCY, complete excision and Thiersch's graft.—Mr. WOOLLCOMBE, partial excision down to eyebrow, and graft, the eyebrow being kept trimmed.

Mr. M. BULTEEL showed a boy, aged 5, who four weeks previously had a fall, striking the right side of his head; bleeding from right ear came on at once and lasted four or five days. There were at no time any symptoms of concussion or compression, but right facial paralysis developed immediately, and has remained. Lately there had been some return of power in tongue.—Mr. LUCY suspected the injury to be in the Fallopian aqueduct, and probably due to hemorrhage. He advised iodides and electricity.

Mr. C. E. Russel Rendle showed a man, aged 27, who nine weeks previously had sustained a compound fracture of lower end of right humerus. Wound now healed and bone united, with good movement, but impaired sensation over ulnar distribution and contraction of little and ring fingers.—Mr. Woollcombe related two similar cases in which the nerve had been successfully dissected out from scar tissue, one case partially relapsing after a time.—Mr. Lucy suggested encasing the nerve in a decalcified bone tube after dissecting out.—Mr. Whiteford explained the cause of the deformity.

Mr. F. E. Row showed a good skiagram of an arm with a double fracture of the olecranon.

Mr. Lucy demonstrated a malignant growth of the sigmoid flexure which had caused intussusception and had been removed *per rectum* successfully.

Mr. Woollcombe showed two uteri removed for malignant disease, one by the vaginal route, and one by the combined abdominal and vaginal method. For malignant disease, when the uterus is not so movable as in health, he preferred the former, using Greig Smith's clamps and not inverting the uterus.

---

*November 3rd*, 1897.

Dr. J. C. Aldridge, in the Chair.

Mr. P. Swain read a paper on "Extra-Uterine Gestation," a report of which will appear in the *Edinburgh Medical Journal*.—This was discussed by Messrs. Russel Rendle, M. Bulteel, Aldous, Rider, Whiteford, Lucy, Hamilton, and Pullen.

W. L. Woollcombe. *Hon. Sec.*

